# Bat-Associated Hemotropic Mycoplasmas in Immunosuppressed Children, Spain, 2024

**DOI:** 10.3201/eid3110.250862

**Published:** 2025-10

**Authors:** Fernando Esperón, Bárbara Martín-Maldonado, Irene Iglesias, Elena Neves, Eleonora Seri, Paula García-Sanchez, Ángel Morillas-Mingorance, Ana Méndez-Echevarría

**Affiliations:** Universidad Europea de Madrid, Villaviciosa de Odón, Spain (F. Esperón, B. Martín-Maldonado, E. Neves, E. Seri); Consejo Superior de Investigaciones Científicas, Valdeolmos, Spain (I. Iglesias); La Paz University Hospital, Madrid, Spain (P. García-Sanchez, Á. Morillas-Mingorance, A. Méndez-Echevarría); Institute for Health Research, Madrid (P. García-Sanchez, A. Méndez-Echevarría); Carlos III Health Institute, Madrid (A. Méndez-Echevarría); CIBERINFEC, Madrid (A. Méndez-Echevarría)

**Keywords:** hemoplasma, hemotropic mycoplasma, bacteria, bats, zoonoses, molecular detection, pediatrics, Spain

## Abstract

We report the detection of hemotropic mycoplasmas in 4 immunosuppressed pediatric patients in Spain: 2 solid organ transplant recipients, 1 hematopoietic stem cell transplant recipient, and 1 cancer patient. Sequences were 100% identical to a strain previously identified in *Miniopterus schreibersii* bats, which raises concerns about unnoticed zoonotic transmission.

Hemotropic mycoplasmas (hemoplasmas) are small, cell-wall–deficient bacteria that attach to erythrocyte surfaces and can induce hemolytic anemia in mammals, particularly in immunocompromised hosts (*1*). Species such as *Mycoplasma hemofelis* in cats and *M. ovis* in sheep are well documented in veterinary medicine (*1*). Human cases, although rare, have been reported in association with immunosuppression or zoonotic exposure (*2*–*4*). Molecular evidence has revealed human infections with multiple hemoplasma species, including *Candidatus* Mycoplasma hematominutum and *M. ovis* (*4*). *Candidatus* Mycoplasma hematohominis, a likely bat-origin species, has been reported in human patients from New Caledonia (*5*), Japan (*6*), France (*7*), and the United Kingdom (*8*); the cases in France and the United Kingdom likely originated in Australia. *Candidatus* M. hematohominis, which causes an illness called flying fox hemolytic fever, is present in *Pteropus* bats; reported overall prevalence is 40% (*5*). Clinical signs in humans typically were anemia, fever, asthenia, abdominal pain, and weight loss (*6*–*9*).

In a previous study (*10*), zoonotic pathogens were investigated in immunosuppressed children and their companion animals in Spain. That cross-sectional study was performed at a national reference center for pediatric transplantation and immunocompromised children. In this study, we included patients <20 years of age in Spain who lived in a house that owned dogs or cats if they had received a solid organ or a hematopoietic stem cell transplantation or if they had an inborn error of immunity or an oncologic or rheumatologic disease for which they were undergoing immunosuppressive treatments or chemotherapy. We contacted patients and families who fulfilled the inclusion criteria by telephone or in person during a visit to the hospital. We used stored blood samples to investigate the presence of hemotropic mycoplasmas by molecular methods. The local Clinical Research Ethics Committee of La Paz University Hospital (PI-4770) approved the study, and all participants or legal guardians provided informed consent.

## The Study

We analyzed 69 EDTA-anticoagulated blood samples collected in 2024 from immunocompromised pediatric patients using real-time PCR targeting a 366-bp fragment of the hemoplasma 16S rRNA gene (*11*). We used sterile deionized water as a negative control; we included an internal positive control to *M. hemocanis*, obtained from a blood sample previously tested and sequenced, in each run. We Sanger sequenced positive samples using both PCR primers and analyzed the sequences by using BLAST (https://blast.ncbi.nlm.nih.gov/Blast.cgi). We constructed a maximum-likelihood phylogenetic tree using MEGA version 12 (https://www.megasoftware.net). The analysis was based on a 329-bp fragment of the 16S rRNA gene (primers excluded), with 500 bootstrap replicates to assess branch support. We selected the Tamura-Nei model with gamma distribution and a proportion of invariant sites as the best-fit nucleotide substitution model in accordance with the Bayesian Information Criterion.

Four samples (5.8% [95% CI 0.3%–11.3%]) tested positive. All 4 were from immunosuppressed children; 1 had chemotherapy-induced bone marrow aplasia (Table). Of the 4 patients, 3 had a history of multiple blood transfusions during their treatment: patient 2 received 3 red blood cell transfusions in 2022; patient 3 received 6 red blood cell and 9 plasma transfusions in 2019; and patient 4 received 30 red blood cell transfusions, 37 platelet pools, and 1 plasma transfusion during 2019–2020. Patient 1 did not receive any blood transfusions. All 4 patients had dogs as companion animals.

**Table:** Characteristics of mycoplasma-positive patients in study of bat-associated hemotropic mycoplasmas in immunosuppressed children, Spain, 2024*

Characteristic	Patient no.			
	1	2	3	4
Age, y/sex	17/F	2/F	4/M	13/M
Region of origin (type)	Zaragoza (urban)	Orense (rural)	Estremera (rural)	Madrid (urban)
Underlying pathology	Papillorenal syndrome, renal transplantation	Rhabdomyosarcoma, chemotherapy	Alagille syndrome, liver transplantation	Acute myeloid leukemia, hematopoietic stem cell transplantation
Mycoplasma detection, Ct	33.95	32.47	33.44	34.17
Blood parameters				
Anemia diagnosis†	No	Yes	No	No
Type of anemia	None	Aplastic anemia‡	None	None
Hb, g/dL	11.8	8.5	12.7	14.9
Hct, %	39.4	26.7	40.8	44.8
Hb after 4 mo, g/dL	11.9	9.3	12.8	14.9
Hct after 4 mo, %	37.4	29	42.2	44
Leukocytes <5,000	No	No	830/mm^3^	No
Splenomegaly†	No	No	No	No
Biochemical parameters				
LDH elevation†	No	No	No	No
Bilirubin elevation†	No	No	No	No

*Ct, cycle threshold; Hb, hemoglobin; Hct, hematocrit; LDH, lactate dehydrogenase. †Hematologic data correspond to the day the PCR-positive blood sample was collected. ‡Secondary effect of chemotherapy.

The sequences from the 4 children’s samples were 100% identical and matched a previously reported strain from Schreiber’s bats (*Miniopterus schreibersii*) in Spain (*12*). We deposited the sequence we isolated into GenBank (accession no. PV698513). BLAST analysis showed 97.5% identity with *Candidatus* M. hemohominis. Phylogenetic analysis clustered those sequences within a clade of bat- and human-associated hemoplasmas (Figure).

**Figure:**
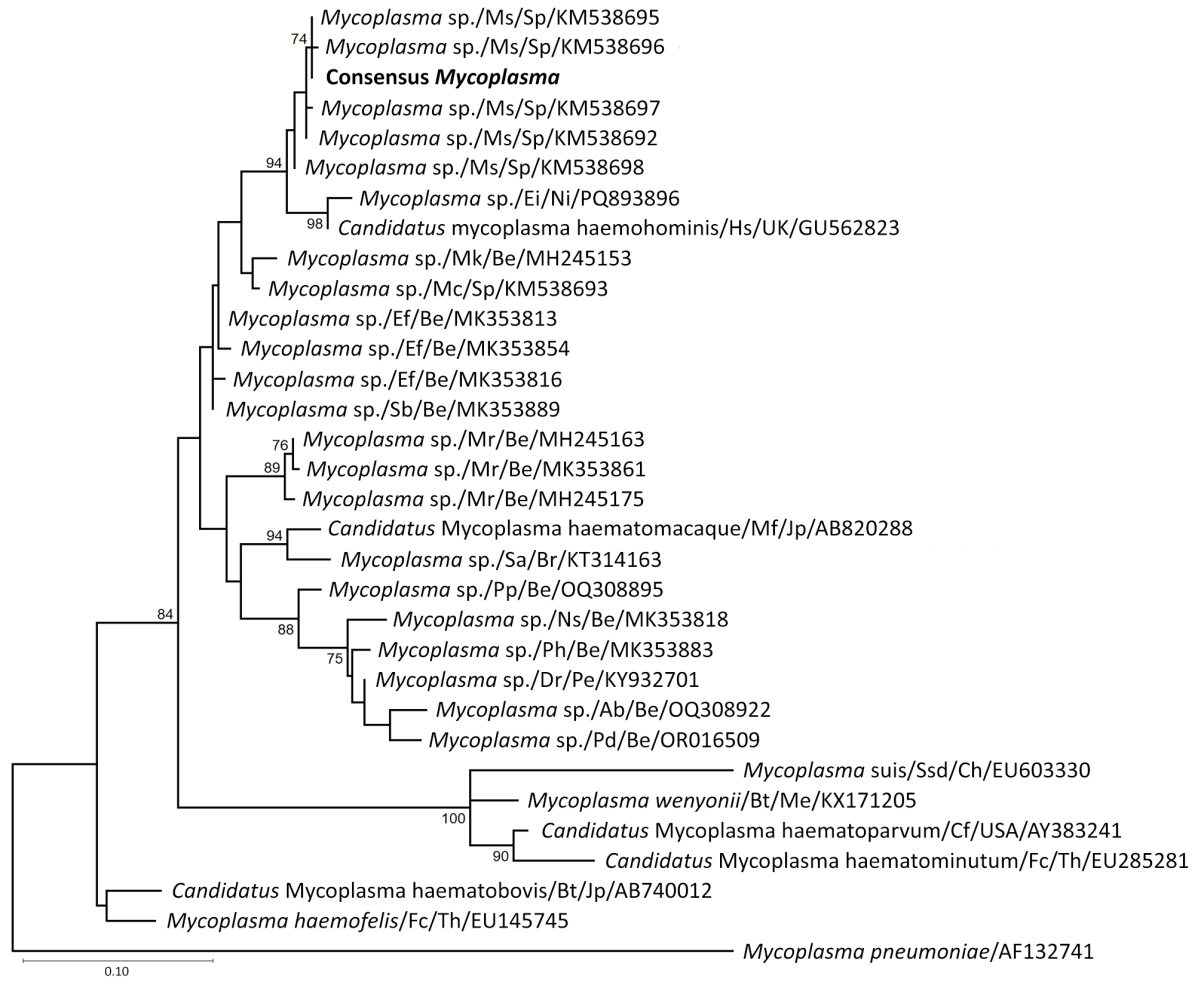
Phylogenetic analysis of sequences from samples collected in 2024 from mycoplasma-positive pediatric patients in study of bat-associated hemotropic mycoplasmas in immunosuppressed children, Spain, 2024. Bold indicates sequence from this study. Numbers at the nodes represent bootstrap support values; values >75% are not shown. GenBank accession numbers are indicated for reference sequences. Scale bar indicates nucleotide substitutions per site. Hosts are indicated as follows: Ab, *Artibeus phaeotis (*pygmy fruit-eating bat); Bt, *Bos taurus* (cattle); Cf, *Canis familiaris* (dog); Dr, *Desmodus rotundus* (common vampire bat); Ef, *Eptesicus furinalis* (Argentine brown bat); Ei, *Eidolon* sp. (palm bat); Fc, *Felis catus* (cat); Hs, *Homo sapiens* (human); Mc, *Myotis capaccinii* (long-fingered bat); Mf, *Macaca fuscata* (Japanese macaque); Mk, *Myotis keaysi* (hairy-legged myotis bat); Mr, *Molossus rufus* (black mastiff bat); Ms, *Miniopterus schreibersii* (bent-wing bat); Ns, *Natalus stramineus mexicanus* (Mexican funnel-eared bat); Pd, *Phyllostomus discolor* (pale spear-nosed bat); Pp, *Pteronotus parnellii* (Parnell's mustached bat); Ph, *Platyrrhinus helleri* (Heller's broad-nosed bat); Sa, *Sapajus apella* (tufted capuchin); Sb, *Saccopteryx bilineata* (greater sac-winged bat); Ssd, *Sus scrofa domesticus* (domestic pig)*.* Countries are indicated as follows: Be, Belize; Br, Brazil; C, China; Jp, Japan; Me, Mexico; Ni, Nigeria; Pe, Peru; Sp, Spain; Th, Thailand; UK, United Kingdom.

Although all 4 patients lived with household pets, we found no evidence that their animals were the source of infection. The main hemoplasma species in dogs is *M. hemocanis*, genetically distinct from the hemoplasmas we detected (*4*). In contrast, the sequences we detected are related to *Candidatus* M. hematohominis, previously associated with bats and humans (*5*–*9*). All other related sequences were from bat hosts. Of note, the observed 97.5% sequence identity is below the levels typically reported among members of the same hemoplasma species, which suggests the existence of a distinct taxon. Longer fragments of the 16S rRNA gene or additional targets such as the *rpoB* gene would improve taxonomic resolution. In our study, the high cycle threshold values (>32) of the positive samples hindered the successful amplification of the full-length gene. Nevertheless, a previous study (*12*) reported consistent classification outcomes when comparing short- and full-length 16S rRNA gene sequences, supporting the hypothesis that the hemoplasmas we detected might represent a distinct species within the broader *M.* *hematohominis*–like clade. The zoonotic potential of this group, including genetically divergent but closely related variants, warrants further investigation.

Unlike previously reported cases that were typically associated with wildlife exposure or rural environments, 2 of our patients lived in urban areas. Moreover, none of the patients had known contact with wildlife or recent travel history, and they lived up to 500 km apart from each other, which suggests not only the possibility of domestic or peridomestic reservoirs or vectors but also that these hemotropic mycoplasmas are widely distributed in the country. Schreiber’s bats are widespread in Spain; they roost primarily in caves, although they are also known to use abandoned buildings, tunnels, and cellars as shelters, which could cause indirect human exposure (*12*). Seasonal movements of Schreiber’s bats can span 300 km. The prevalence of hemoplasmas in this species has been reported as high as 97% (*12*). Those factors could explain the detection of the hemoplasma in children living 500 km apart. Because studies have detected 16S rRNA gene sequences with high (>99.5%) nucleotide identity to known hemoplasmas in ectoparasites such as ticks and sand flies collected from bats (*9*,*13*), vectorborne transmission of hemoplasmas has been hypothesized; however, our patients had no reported arthropod contact. Previous cases of *M. ovis* and *Candidatus* M. hematoparvum infection in humans also lacked known arthropod exposure (*14*), indicating potentially complex transmission routes.

All hemoplasma-positive cases in our study were in severely immunocompromised children, a group particularly vulnerable to emerging infections. Although direct clinical consequences were unclear, 1 patient experienced bone marrow aplasia, likely attributable to chemotherapy.

## Conclusions

Hemoplasmas are known to persist in the bloodstream for extended periods (*15*). Although we observed no direct clinical effect in this study, the unexpected detection of bat-related hemoplasmas in immunosuppressed patients raises questions about their potential for transfusion-associated or vectorborne transmission. We emphasize that PCR detection alone does not indicate viability. In addition, the 4 positive samples showed high cycle threshold values, suggesting low DNA copy numbers compatible with trace levels or nonviable infections. Because we did not test organ, hematopoietic stem cell, or blood donors related to the patients, we cannot assess the possibility of transfusion- or transplant-associated transmission. Further investigation could determine whether these organisms can withstand blood processing and storage, and whether they might pose any risk for transmission under specific conditions.

In summary, our study presented molecular evidence of bat-related hemotropic mycoplasmas in immunosuppressed children from Spain who have pets in a setting without reported wildlife contact or travel history. Our findings highlight the need for increased surveillance of emerging zoonotic infections, especially in high-risk populations.
